# Characterisation and evaluation of physical properties of AH-Plus sealer with and without the incorporation of petasin, pachymic acid, curcumin and shilajit-an invitro study

**DOI:** 10.1186/s12903-024-04108-w

**Published:** 2024-03-19

**Authors:** Selvanathan MJ Vinola, Sekar Mahalaxmi

**Affiliations:** Department of Conservative Dentistry and Endodontics, SRM Dental College, Bharathi Salai, Chennai, Tamil Nadu 600089 India

**Keywords:** AH plus sealer, Curcumin, Characterisation of resin sealers, Natural compounds, Pachymic acid, Petasin, Shilajit, Physical properties, Phytochemicals, Root canal sealers

## Abstract

**Background:**

AH Plus, an epoxy resin-based sealer, is widely used in endodontic practice, owing to its good physical properties that confers longstanding dimensional stability and good adhesion to dentin. Nevertheless, its propensity to trigger inflammation, especially in its freshly mixed state, has been extensively documented. Phytochemicals such as Petasin, Pachymic acid, Curcumin, and Shilajit are known for their anti-inflammatory and analgesic effects. This study aimed to analyze and determine the effect of these natural products on the physical properties of AH Plus sealer when incorporated with the sealer.

**Methods:**

AH Plus (AHR) sealer was mixed with 10% petasin, 0.75% pachymic, 0.5% and 6%shilajit to obtain AHP, AHA, AHC and AHS in the ratio of 10:1 and 5:1 respectively. Five samples of each material were assessed for setting time, solubility, flow, and dimensional stability in accordance with the ISO 6876:2012 standardization. Sealers were characterized through scanning electron microscopy (SEM), X-ray energy dispersive spectroscopy, X-ray diffraction (XRD), and Fourier transform infrared (FTIR) spectroscopy. Statistical evaluation involved the Kolmogorov-Smirnov and Shapiro-Wilks tests for normality and the one-way ANOVA test for analysis.

**Results:**

In this investigation, the characterisation analysis revealed a relatively similar microstructure in all the experimental root canal sealers. All experimental groups, excluding the control group, exhibited an increase in flow ranging from 11.9 to 31.4% at a 10:1 ratio. Similarly, for the 5:1 ratio, the increase ranged from 12.02 to 31.83%. In terms of dimensional stability, all groups at the 10:1 ratio showed a decrease compared to the control group. The addition of natural agents to AHR in 10:1 ratio led to a reduction in setting time by 8.9–31.6%, and at a 5:1 ratio, the reduction ranged from 8.1 to 31.5%. However, regarding solubility, the addition of natural agents did not induce any significant alterations.

**Conclusion:**

Based on the results of this study, it can be concluded that all tested root canal sealers exhibited properties that met the acceptable criteria outlined in the ISO 6876:2012 standardization.

## Background

The success of an endodontic treatment hinges on thorough root canal debridement, complemented by three-dimensional obturation using a core material and sealer that are both dimensionally stable and biocompatible [1, 2]. Gutta-percha is the most commonly utilized core material in this context. The necessity for root canal sealers (RCSs) arises from the limitation of gutta-percha to adhere to the root canal walls and effectively seal the anatomic irregularities of the canal. RCSs are available in various types, classified based on their chemical composition. These include zinc oxide eugenol-based, calcium hydroxide-based, glass ionomer-based, resin-based, and bioceramic sealers [3, 4]. Among these, AH Plus resin sealer (AHR; Dentsply DeTrey GmbH, Konstanz, Germany), a two-paste system is widely used and extensively studied. AHR is often regarded as the “gold standard” sealer due to its excellent adhesion to dentin, biocompatibility, and favorable physical properties [4, 5]. However, if the sealer is inadvertently extruded into the periapical region through the apical foramen, studies have indicated that the cytotoxic effects tend to be higher before the sealer sets [6–8]. Sousa CJ et al. (2006) stated that toxicity induced by sealers plays a significant role in initiating destruction at the periapical tissues which in turn, has a substantial impact on alveolar bone resorption, ultimately leading to a poor prognosis [9].

Since the introduction of AHR, ongoing research has focused on modifying it with various agents such as Hinokitiol, amoxicillin, chlorhexidine, Cetrimide, calcium hydroxide, simvastatin and others [10–15]. Indeed, phytochemicals like petasin, pachymic acid, curcumin, and shilajit have garnered attention for their significant anti-inflammatory properties. Petasin, derived from the Petasites hybridus (butterbur) plant, is well-documented for its wide-ranging anti-inflammatory and analgesic effects, and its applications in the treatment of various conditions, including migraines, urogenital inflammations, digestive tract infections, asthma, allergic rhinitis, allergic skin disease, gastric ulcers, ocular allergy, and wounds [16, 17]. Pachymic acid, a triterpenoid extracted from the fruiting body of Fomitopsis nigra mushrooms, has demonstrated anti-inflammatory properties and is non-cytotoxic when added to AHR as demonstrated by Arun S et al. (2017) and various authors [8, 18, 19]. Curcumin, known chemically as 1,7-bis(4-hydroxy-3-methoxyphenyl)-1,6-heptadiene-3,5-dione, possesses anti-inflammatory, antioxidant, anti-tumor, and various other biological activities [20–22]. Shilajit (asphaltum) is produced by the long term humification of dead plant material and organic vegetable matter by different micro-organisms and has great potential for the treatment of a variety of human conditions as reviewed by Besedovsky, exerting an influence on endocrine, autonomic, and brain functional changes [23–25].

Previous studies by various authors using these phytochemicals individually have assessed their physicochemical properties and optimized the concentrations of 15% petasin, 0.5% pachymic acid, 1% curcumin and 4% shilajit respectively [8, 17, 21, 25]. In case of petasin-modified zinc oxide eugenol sealer (ZOEs), evaluations of setting time and solubility revealed that the addition of petasin in a 5:1 ratio significantly reduced the setting time of ZOEs, although it did not markedly affect the solubility of the sealer [17]. The physico chemical properties of resin based sealer incorporated with pachymic acid did not alter significantly and were found to be within the standards outlined by ISO 6876:2012 [8]. Sahebalam et al. explored the solubility of zinc oxide eugenol combined with varying concentrations of nano-curcumin. Their findings indicated that solubility increased with higher curcumin concentrations [20]. Sarah et al. evaluated the cytotoxicity and antiinflammatory properties of shilajit nutraceutical and concluded that it can be a viable alternative to conventional antiinflammatory drugs in the field of medicine and dentistry [25].

With the above evidenced research, the incorporation of either of these phytochemicals to AHR sealer could be an effective modification that may subdue the detrimental effects caused by it. However, any additive to the existing composition of AHR will alter the formulation of the original sealer. Though modifications with individual components have been attempted so far, there are very few studies that give a detailed analysis of the physicochemical properties of the modified AHR. To further optimize the appropriate concentration of the phytochemical to be added to the sealer, a pilot study was conducted with the above mentioned concemtrations along with a higher and lower concentration. The concentration with the most optimal setting time and dimensional stability were considered for this study. Hence the concentrations of 10% petasin, 0.75% pachymic acid, 0.5% Curcumin and 6% Shilajit was used for this study.

The current study was thus aimed to characterise and investigate the setting time, solubility, flow and dimensional stability of AHR with and without incorporation of petasin, pachymic acid, curcumin and shilajit. In the present study, setting time, solubility, flow and dimensional stability of AHR and its modifications were measured as outlined in the International Standard ISO 6876:2012 for dental root canal sealing materials [26]. The null hypothesis was that, the incorporation of these phytochemicals to AHR shall not significantly change the above mentioned properties.

## Methods

The research protocol was presented to Institutional Review Board (IRB) at SRM institute of science and technology and approval was obtained **SRMDC/IRB/2019/PhD/No.112.**

### Preparation of the experimental materials

Conventional sealer AHR (Dentsply DeTrey in Konstanz, Germany Lot No: 2,204,000,072) was mixed according to the manufacturer’s instructions. 0.l mL of 10% petasin (Vedic, Noida, India Lot No: 244,001) solution was mixed thoroughly with the 0.9 mL of freshly mixed AHR sealer to obtain AHP; similarly 0.1mL each of 0.75% pachymic acid (Bio Corporals Co., Ltd, Chennai, India, Lot No: 113,870,974), 0.5% curcumin (High Purity Laboratory Chemicals Pvt. Ltd., Mumbai, India, Lot No: K085/0241120II09) and 6%shilajit (Kishore Pharma Ltd, Punjab, India, Lot No:) was mixed with 0.9 mL of AHR to obtain AHA, AHC and AHS in the ratio of 10:1. Similarly each 0.2mL phytochemical was mixed with 0.8mL of AHR in the ratio 5:1 respectively.

All the groups except the conventional sealer group AHR were designated with 10 or 5 depending on the specific ratio (10:1 or 5:1) of the modified sealer. For example, AHP5, AHP10 etc.

### Evaluation of physical properties

The sample size was determined using G*power version 3.0. According to the parent article, the sample size was calculated using One- way ANOVA, keeping the effect size as 1.13 with an alpha error level of 0.05 and achieving a power of 0.90, the total sample size was as *N* = 20, with *n* = 5 for each group. The following physical properties were evaluated according to ISO 6876:2012 standardisation.

#### Setting time

The experimental sealers were manipulated and placed inside polytetrafluoroethylene molds with an internal diameter of 10 mm and a height of 2 mm. The molds were positioned on a glass plate, and the sealer was filled up to the brim. This entire setup was then stored in a cabinet at 37 ± 1 °C and > 95% relative humidity. The experiment was started as the manufacturer’s specified setting time of 8 h approached, wherein a Vicat’s indenter—weighing 100 ± 0.5 g and equipped with a flat-ended needle tip with a diameter of 2 ± 0.1 mm was brought into contact with the sealer surface. This process was repeated at a new location every hour up to 20 h and then at five-minute intervals until no further indentations was observed. The procedure was stopped when indentations became invisible on the sealer surface. The time elapsed from the initiation of sealer manipulation until the point where no indentations were produced was recorded as the setting time for that specific sample. The mean of these recorded times was then considered as the setting time for the experimental sealer.

#### Solubility

The sealers were mixed and placed into polytetrafluoroethylene molds with a diameter of 20 ± 1 mm and a height of 1.5 ± 0.1 mm and allowed to set in an incubator maintained at 37 °C under 95% relative humidity. Once set, the sealer samples were removed from their molds, weighed using an analytical balance (W1), and then immersed in test tubes containing 10 mL of distilled water. The samples were then retrieved at 1, 3, 7, and 14 days, and dried using absorbent paper and stored inside a desiccator. Following the drying process the samples were weighed again (W2). The solubility (S) was calculated using the formula.

$${\rm{S}}\,{\rm{ = }}\,\left( {{\rm{W1}}\,{\rm{-}}\,{\rm{W2}}} \right){\rm{/W1}}\, \times \,{\rm{1}}00$$

The mean value was taken as the solubility of that sample.

#### Flow

Using a 1 mL graduated disposable syringe, 0.05 ± 0.005 mL of the mixed sealer was dispensed onto a glass plate. Approximately 3 min from the onset of manipulation, another glass plate was centrally placed on top of the sealer, and a weighing stone of 100 g was positioned to maintain a total mass of 120 ± 2 g. The weight was removed after 10 min from the beginning of manipulation. Using a digital vernier caliper with a resolution of 0.01 mm, the largest and smallest diameters of the compressed sealer disc were measured. The mean of these diameters was recorded as the flow value when the diameters were within 1 mm of each other. If not, the test was repeated. This experiment was conducted three times, and the mean was calculated. The result was then corrected to the nearest integer and documented as the flow in millimeters.

#### Dimensional change

The mixed material was placed into cylindrical silicon molds with an internal diameter of 6 mm and a height of 12 mm (*n* = 5). Once set, the distance between the flat ends (M1) was measured with an accuracy of 10 μm using a digital caliper. Subsequently, the materials were stored in distilled water at 37 ± 1 °C. Similar measurements of the distance (M2) were taken again after 7, 14, and 21 days. The test was repeated three times, and the mean change in length was calculated as the dimensional change (D) using the formula, D= (M2 – M1)/M1 × 100.

### Characterisation

#### Surface morphology and elemental analysis

Disc-shaped specimens (10 mm in diameter and 2 mm high) were prepared and immersed in Hank’s Balanced Salt Solution (HBSS) for a total of 28 days. Following immersion, the samples were taken out and placed in acetone for 48 h, and dessicated in a vacuum desiccator for an additional 24 h. Subsequently, the surfaces of the samples were ground with progressively finer diamond discs using an automatic polishing machine. Both control and experimental groups were coated with carbon using a CC7650 SEM Carbon Coater Unit (Quorum Technologies Ltd.). The superficial surface morphology and the element distribution of the coated discs was individually examined at two time intervals of one day and 28 days, using a scanning electron microscopy (SEM) unit (Jeol 6100 EDAX; Jeol Inc.) at 150X magnification, coupled with an energy-dispersive spectroscopy (EDS) system (INCA 350 EDS; Oxford Instruments) for elemental analysis.

#### Phase identification of crystalline material

The set experimental sealers were crushed to a fine powder using a mortar and pestle. Phase analysis was conducted using a Bruker D8 diffractometer. X-ray patterns were acquired with a step size of 0.02 and a duration of 0.6 s per step. Phase identification was carried out using search-match software with the International Centre for Diffraction Data database (ICDD) to assess the composition and identify the phases present in the material.

#### Analysis of functional groups

A FTIR spectrometer (model 8700; Shimatzu, Tokyo, Japan) equipped with a micro-MIR cell was utilized under the following conditions: a range of 4,000 to 400 cm^-1, a resolution of 4 cm^-1, 60 scans co-addition, and a 45° angle edge KRS-5 crystal (10 × 5 × 1 mm) with 7 internal reflections is used in this study to analyze the presence of functional groups at a depth of 3 μm at 1,000 cm^-1. Throughout the storage period, all specimens were maintained at 37 °C and 100% relative humidity. Set materials were pressed against the KRS-5 crystal using the torque-wrench device of the micro-MIR cell. The reaction rate was calculated from the peak absorbance area. The reaction rates of all the tested materials were expressed as a percentage to assess the different functional groups present in the given sample.

### Statistical analysis

For the physical properties, namely setting time, solubility, flow, and dimensional stability, normality tests such as Kolmogorov-Smirnov and Shapiro-Wilks indicated that the study data followed a normal distribution. Consequently, parametric tests were applied for data analysis. Descriptive statistics were employed to assess the mean among the study variables. Inferential statistics involved the use of one-way ANOVA tests to evaluate comparisons within groups, while unpaired t-tests were performed for comparisons between two groups. The data were analyzed using SPSS (IBM SPSS Statistics for Windows, Version 26.0, Armonk, NY: IBM Corp. Released 2019). The significance level was set at 5% (α = 0.05), with a p-value < 0.05 considered statistically significant.

## Results

### Characterisation analysis

**Fig. 1:**
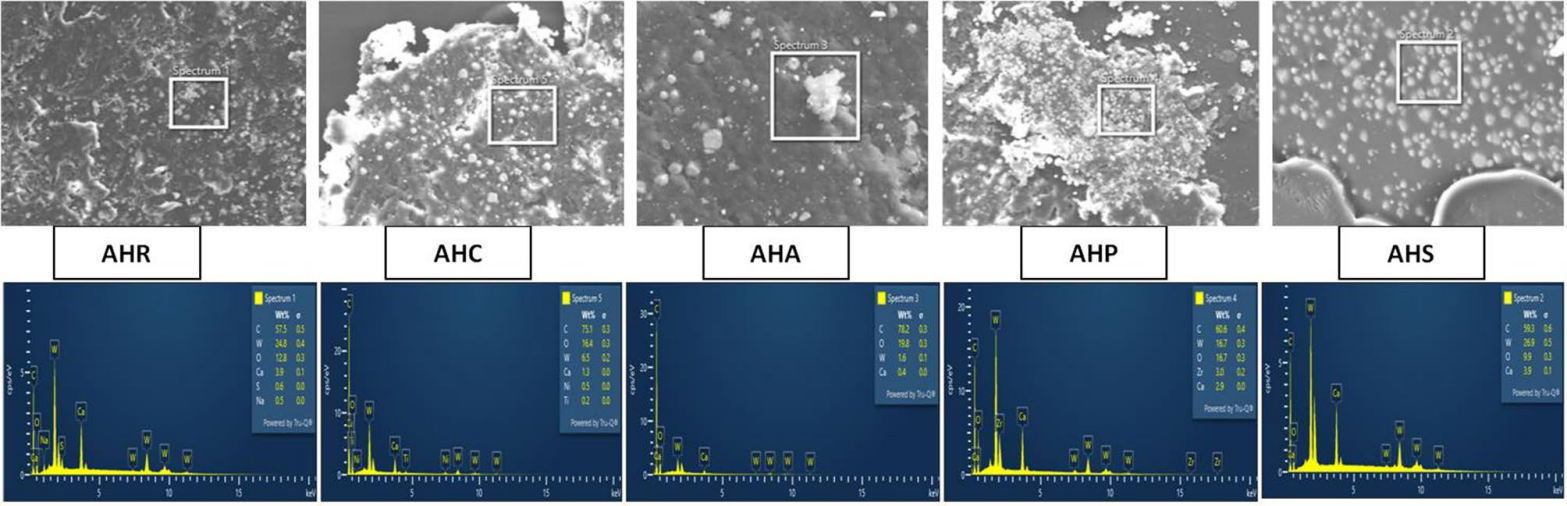
(**a**) The scanning electron micrographs of the experimental samples at 150× magnification. (**b**) EDX microanalysis graphs of the experimental samples

The SEM image of control group (Fig. 1a) exhibits distinctive surface morphology with crystalline structures. AHP and AHC showed clusters on the surface with AHS showing presence of globules. Addition of pachymic acid has produced a smoother surface similar to that of AHR. Quantitative results of elements obtained through EDX microanalysis revealed the presence of carbon, oxygen, tungsten, and calcium in all the tested groups (Fig. 1b). The additional elements depicted by the different groups included sulfur and sodium (control group), zirconium (AHP), nickel and titanium (AHC). Notably, none of the tested sealers contained bismuth or other heavy metals such as lead, chromium, cobalt, copper, zinc, and manganese.

**Fig. 2:**
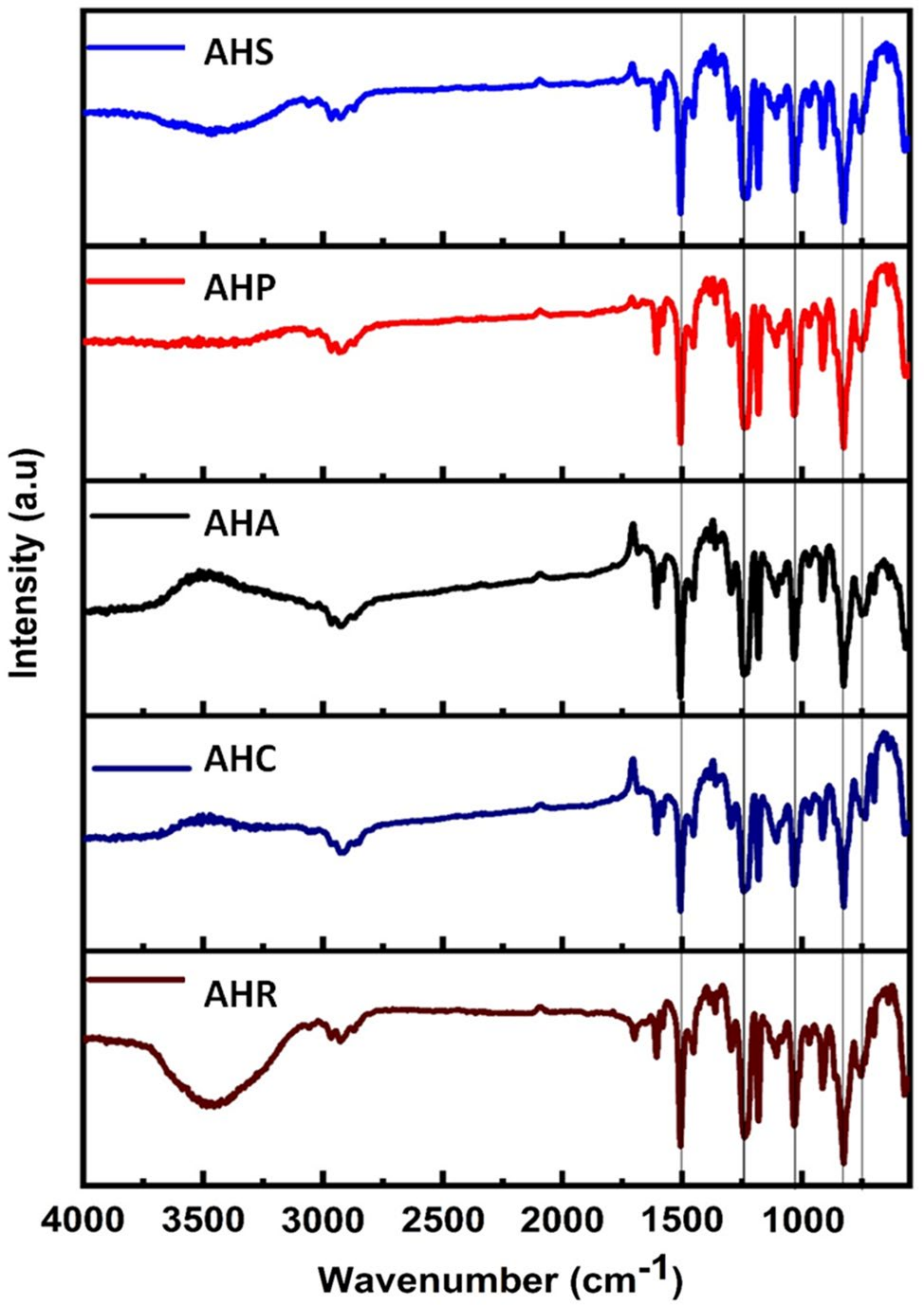
The FTIR spectrum of the experimental sealers revealing strong absorption bands between 749 ^cm−1^ and 1608^cm − 1^

The FTIR spectra of AHR and the experimental sealer reveal strong absorption bands at approximately 749 ^cm−1^, 829 ^cm−1^, 918 ^cm−1^, 1037 ^cm−1^, 1179 ^cm−1^, 1249 ^cm−1^, 1509 ^cm−1^, and 1608 ^cm−1^ (Fig. 2). These bands correspond to specific functional groups, including the -C-H bending group indicative of the compound class strong 1,2 disubstituted (cis) or monosubstituted benzene derivative, -C-H bending group denoting strong 1,4 disubstituted or 1,2,3,4-tetrasubstituted compound class, -C = C- bending group indicating the presence of monosubstituted alkene, -S = O stretching group corresponding to the presence of sulfoxide, medium stretching -C-N denoting the presence of an amine, strong aromatic ester in the form of -C-O stretching, strong stretching of -N-O indicating the presence of nitro compounds, and stretching of -C = O denoting the presence of α-β-unsaturated ketone. Apart from this, there is transmittance peak 3459 cm-1 corresponding to -OH stretching presented in the AHR. In case of AHA and AHC there is transmittance peak at 1701 cm-1 corresponding to conjugated carbon group. While comparing to other samples these peak intensities is low and confirms the absence of new functional groups present in any of the experimental sealers.

**Fig. 3:**
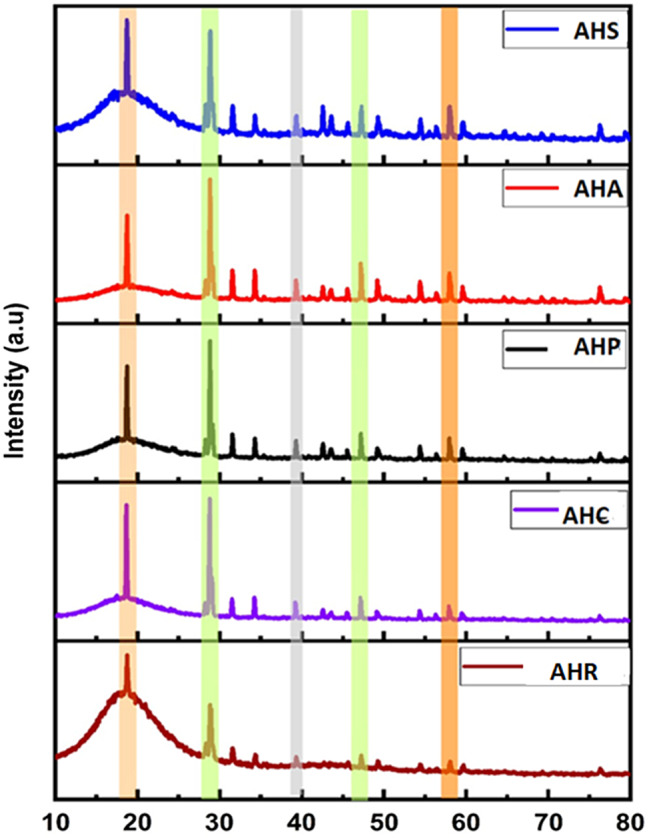
The peaks of XRD diffraction plots of experimental sealers measured in the θ-2θ range

The peaks in the X-ray diffraction (XRD) patterns, as illustrated in Fig. 3, were measured within the 2θ range scale from 10 to 80°. The measurements were conducted using Cu Kα1 radiation at a scan rate of 0.02°/s. The presence of 11.4% of crystalline and 88.6% of amorphous components was present in AHR. In AHP there is presence of 39.9% of crystalline and 60.1% of amorphous components with 50.9% of crystalline and 49.1% of amorphous components in AHA, 36.5% of crystalline and 63.5% of amorphous components and in AHS with 32.6% of crystalline and 67.4% of amorphous components. The obtained results match with previous reference of Joint Committee on Powder Diffraction Standards (JCPDS) [96-152-8786].The presence of Calcium Tungsten Oxide was present in all the experimental groups. There are no new compounds formed in the new sealers. Thus, the addition of these phytochemicals does not alter the composition of the sealer since no new compounds are formed.

### Physical properties

The mean values with standard deviation for dimensional stability, solubility, setting time and flow are given in Tables 1 and 2 for all the groups at different time intervals and different ratios.

#### Setting time

**Fig. 4:**
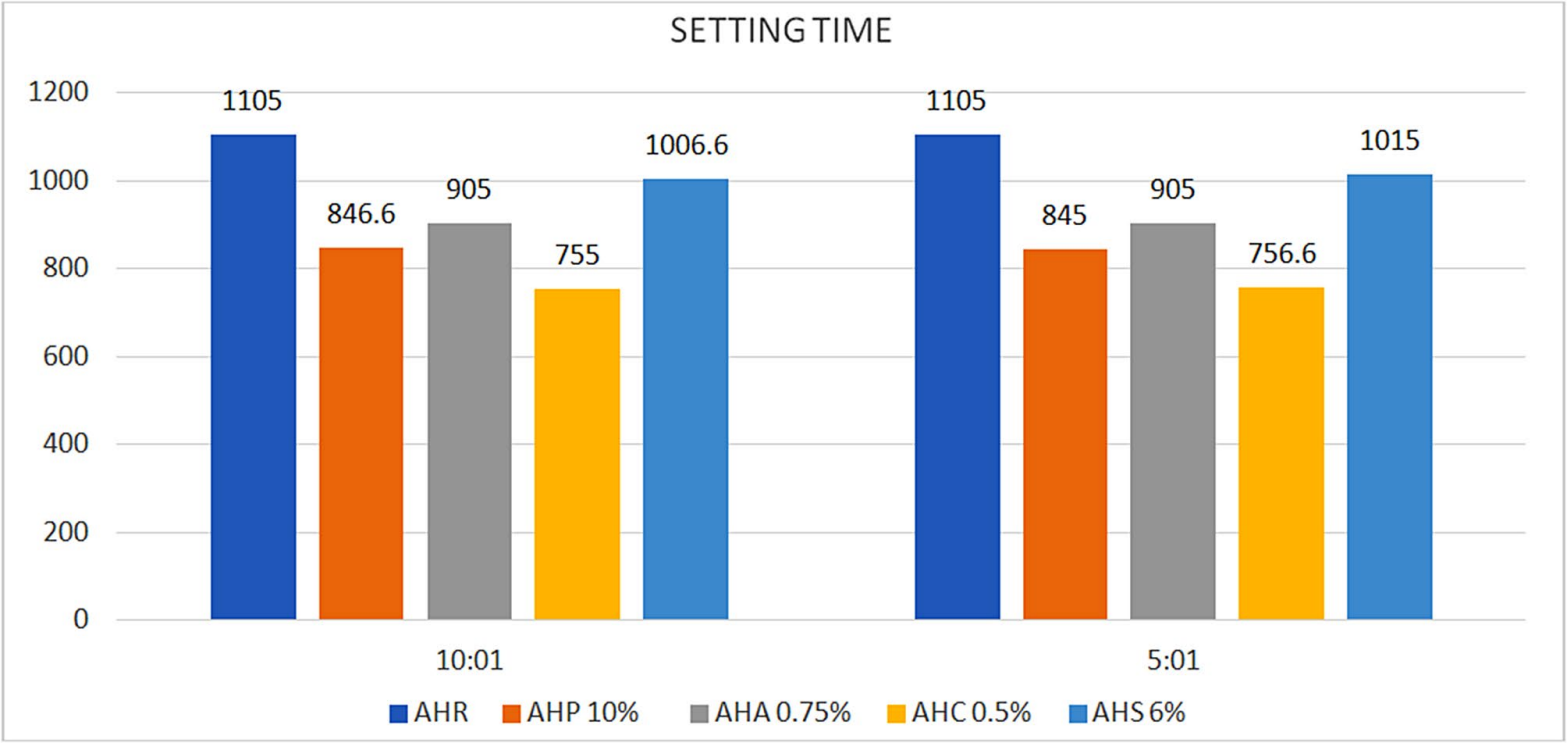
Bar diagram – comparison of mean setting time (min)

The average setting time of conventional AHR is 1105 min. Addition of phytochemicals to AHR in a 10:1 ratio resulted in reduction of setting time by 8.9–31.6%. In the case of 5:1 ratio, experimental samples demonstrated a decrease in setting time ranging from 8.1 to 31.5% (Fig. 4). Specifically, for groups AHP, the experimental samples in 5:1 ratio showed decreased setting time compared to 10:1 ratio, while for AHC and AHS, the trend was vice versa. Notably, AHA values remained the same. However, intergroup comparison revealed statistically insignificant differences in setting time values.

#### Solubility

**Fig. 5:**
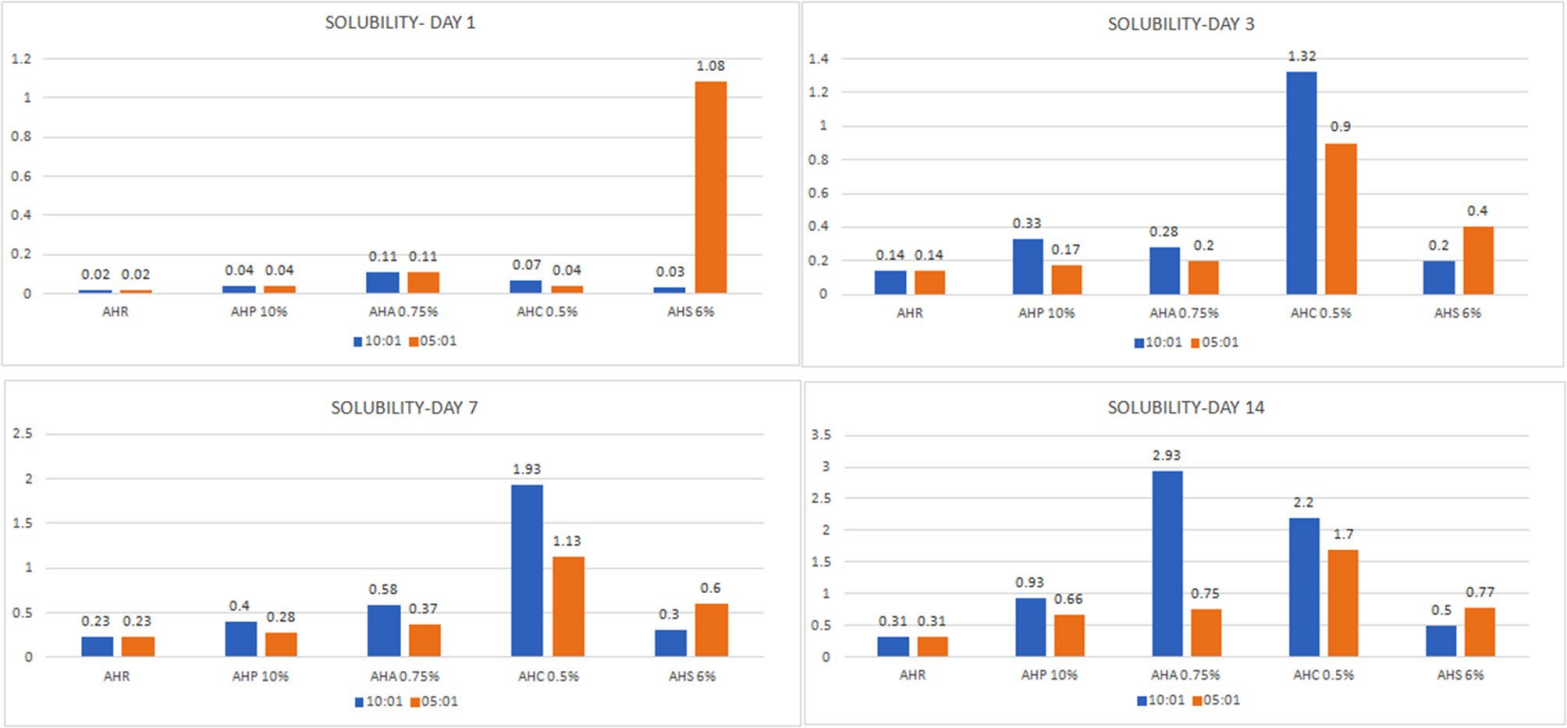
Bar diagram – comparison of mean solubility values of 1, 3, 7 and 14

The solubility of curcumin group was higher compared to the other groups, though not statistically significant (Fig. 5). For the ratio 10:1, on days 1, 3, 7 and 14 there was a gradual percentage increase in solubility. Addition of phytochemicals did not alter the solubility of the AHR sealer.

#### Dimensional stability

**Fig. 6:**
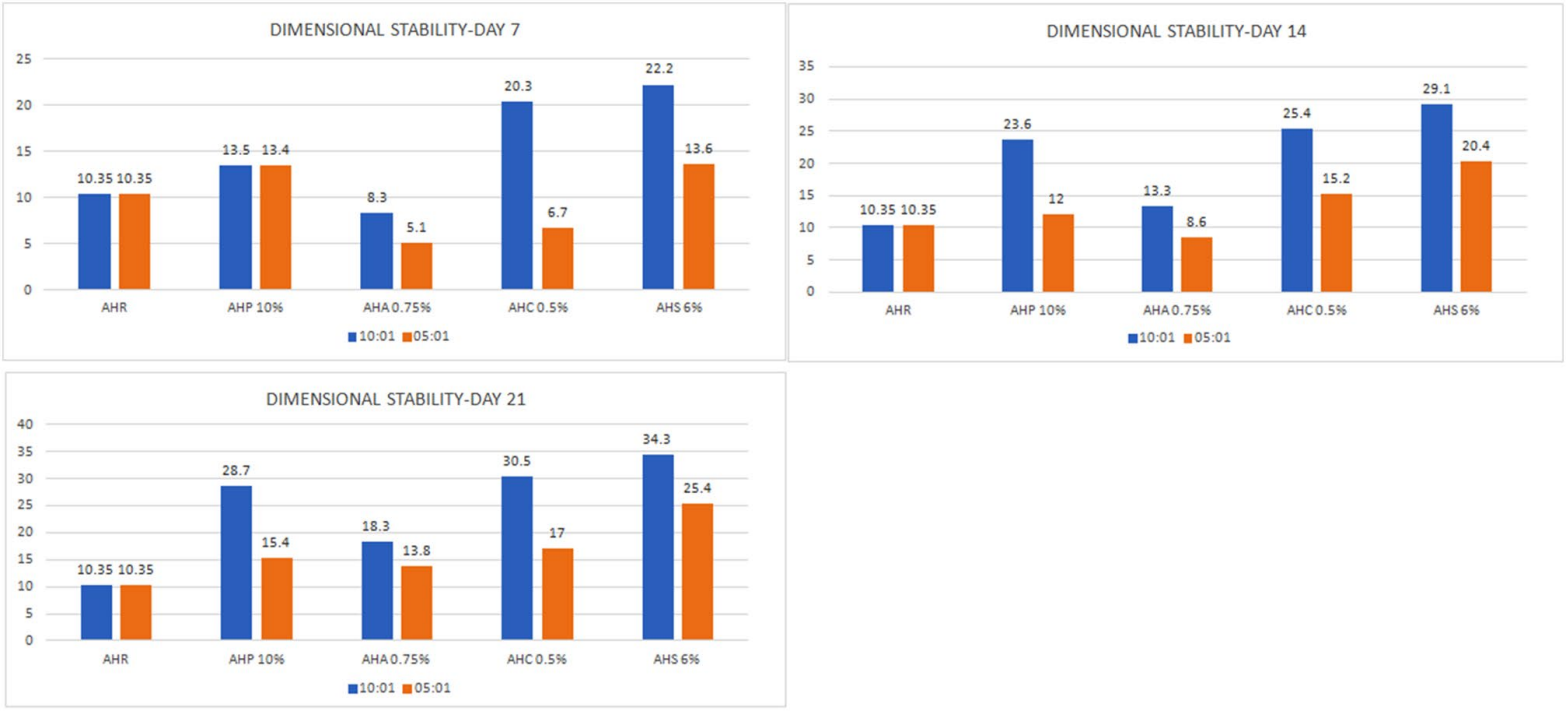
Bar diagram – comparison of Dimensional stability Day 3, 7, 14 and 21

Compared to the control group, all the other groups of 10:1 ratio showed a decrease in the dimensional stability at day 7 that gradually reduced at day 21 (Fig. 6). The experimental samples in ratio 5:1 showed decreased dimensional stability compared to 10:1. The intergroup comparison showed statistically insignificant values.

#### Flow

**Fig. 7:**
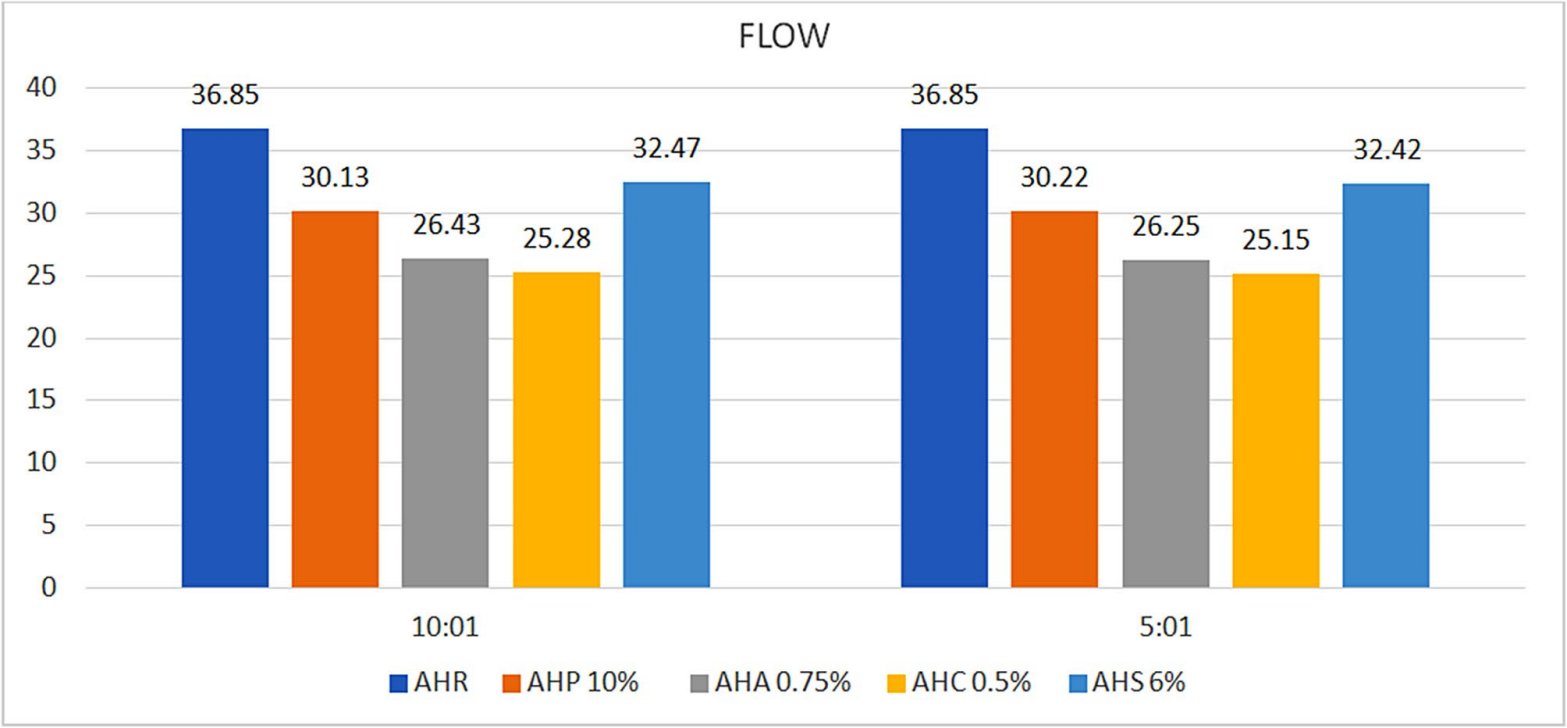
Bar diagram – comparison of mean flow values

All the experimental groups other than the control group, showed an increase in flow from 11.9 to 31.4% at 10:1 ratio, while for the 5:1 ratio, the increase was from 12.02 to 31.83% (Fig. 7). The experimental samples in ratio 5:1 showed decreased flow compared to 10:1. The intergroup comparison showed statistically insignificant values.

**Table 1:** The mean values and standard deviation of dimensional stability, solubility, setting time and flow of all the samples for both the ratios at different time intervals for 10:1 ratio

Groups 10:1	DIMENSIONAL STABILITY			SOLUBILITY				SETTING TIME	FLOW
	DAY 7	DAY 14	DAY 21	DAY 1	DAY 3	DAY 7	DAY 14		
AHP 10%	13.5 ± 2.4	23.6 ± 3.9	28.7 ± 3.1	0.04 ± 0.003	0.33 ± 0.07	0.4 ± 0.004	0.93 ± 0.01	846.6 ± 102.1	30.13 ± 5.2
AHA 0.75%	8.3 ± 1.3	13.3 ± 2.4	18.3 ± 2.2	0.11 ± 0.024	0.28 ± 0.02	0.58 ± 0.03	2.93 ± 0.09	905 ± 121.4	26.43 ± 3.5
AHC 0.5%	20.3 ± 2.4	25.4 ± 1.5	30.5 ± 5.7	0.07 ± 0.002	1.32 ± 0.3	1.93 ± 0.12	2.2 ± 0.4	736.6 ± 93.8	25.28 ± 3.9
AHS 6%	22.2 ± 3.5	29.1 ± 1.9	34.3 ± 3.4	0.03 ± 0.001	0.2 ± 0.01	0.3 ± 0.006	0.5 ± 0.009	1006.6 ± 128.3	32.47 ± 3.1

**Table 2:** The mean values and standard deviation of dimensional stability, solubility, setting time and flow of all the samples for both the ratios at different time intervals for 5:1 ratio

Groups 5:1	DIMENSIONAL STABILITY			SOLUBILITY				SETTING TIME	FLOW
	DAY 7	DAY 14	DAY 21	DAY 1	DAY 3	DAY 7	DAY 14		
AHP 10%	13.4 ± 2.1	12 ± 2.3	15.4 ± 2.4	0.04 ± 0.001	0.17 ± 0.015	0.28 ± 0.011	0.66 ± 0.011	845 ± 42.8	30.22 ± 3.4
AHA 0.75%	5.1 ± 1.3	8.6 ± 1.5	13.8 ± 2.8	0.11 ± 0.021	0.2 ± 0.011	0.37 ± 0.041	0.75 ± 0.001	905 ± 48.1	26.25 ± 3.1
AHC 0.5%	6.7 ± 1.4	15.2 ± 1.1	17 ± 2.1	0.04 ± 0.014	0.9 ± 0.0041	1.13 ± 0.015	1.7 ± 0.23	746.6 ± 41.4	25.15 ± 2.8
AHS 6%	13.6 ± 2.8	20.4 ± 2.8	25.4 ± 4.5	1.08 ± 0.2	0.4 ± 0.019	0.6 ± 0.051	0.77 ± 0.041	1015 ± 203.5	32.42 ± 4.1

## Discussion

Root canal sealers (RCSs) play a crucial role in endodontic therapy by sealing the root canal GP interface, thereby preventing recurrent infection and promoting healing of periapical tissues [27]. In their freshly mixed state, most of the sealers elicit inflammatory reaction in the periapical region [28, 29]. Addition of modifiers to the sealer influence their physical, chemical, and biological properties and understanding these factors is essential for their effective use in clinical practice. Thus this study was designed with the objective of characterizing and evaluating the physical properties of the resin sealer modified with various phytochemicals. The phytochemicals target various inflammatory mediators such as cyclooxygenase-2, inducible nitric oxide synthase, and nuclear factor κB (NF-κB), thereby attenuating the release of proinflammatory and profibrotic cytokines, and suppressing chronic production of free radicals, which culminates in the amelioration of tissue toxicity [30–32].

As stated by Vinola et al., and various authors 15% petasin demonstrated potent anti-inflammatory, analgesic, and anti-spasmodic properties attributed to its sequiterpene content [17, 33, 34]. Regarding pachymic acid, Arun et al. found that 0.5% exhibited profound anti-inflammatory effects by acting on reactive oxygen species (ROS) formation, thereby reducing sealer-induced cytotoxicity [8]. Jacob et al. reported that 1% curcumin exerts anti-inflammatory effects mediated by the upregulation of peroxisome proliferator-activated receptor-gamma (PPAR-γ) activation [21, 35]. Additionally, 4% shilajit, containing the biologically active compound fulvic acid, is recognized for its therapeutic properties, including antioxidant, anti-inflammatory, anti-allergic, immunomodulatory, antidiabetic, anxiolytic effects, and cognitive enhancement, with positive interactions with other drugs [24, 36]. A pilot study was conducted for these materials, examining three different concentrations for each, including an upper limit concentration, a lower limit, and a middle limit based on literature. The best-optimized concentration from the pilot study was then selected for this study. To ensure that the material does not alter the constituents of the original sealers, minimum concentrations were added to the AHR. Thus the study investigated two different ratios, 10:1 and 5:1, to determine the most effective ratio.

Setting time is a critical factor in the clinical application of sealers, as it influences the working time during the procedure and the sealers’ physical properties, including their solubility and dimensional stability [37]. A significant limitation of AHR sealer is its extended setting duration, which surpasses that of other sealers. However our results suggest that though 5:1 ratio facilitated faster setting for AHP, while 10:1 ratio led to quicker setting for AHS and AHC, with AHA showing the same setting time in both ratios (Fig. 4). The addition of phytochemicals did not improve the setting time of the sealer to a clinically acceptable range, which may be considered as a limitation of the study. When a sealer remains unset or only partially sets, there is an increased risk of more rapid infiltration by irritants and their byproducts to the periapical region [38].

When compared to the previous studies there is variation in the setting time of AHR in this study, which is less, compared to the study done by Preethi et al. (2020) whereas it is the same compared to the study done by Koo et al. (2023) [39, 40]. The observed variability might be attributed to variation in mold used, handling, temperature, relative humidity and manipulation of the sealer. The setting time using a stainless-steel mold was significantly longer than using gypsum mold (Koo et al. 2023) [39]. Non uniform dispensing of the components within the tube, specific segment of the tube in use, may also influence the result [41]. Furthermore, the values obtained fall within the 10% variation range deemed acceptable by ANSI/ADA standards. Given that there is no statistically significant difference between the ratios, either the 10:1 or 5:1 ratio can be utilized for the modified sealer, though a lower concentration and volume of the modifier is preferred.

The solubility of the sealers not only affects their degradation rate but also has implications on their biocompatibility, owing to the leaching of the components into the periapical tissues [42]. It also influences their long-term stability and performance within the root canal system [43, 44]. Our results suggest that the solubility of all experimental sealers was higher than that of the control, but these differences were statistically insignificant. Notably, the groups with 5:1 ratio exhibited lower solubility compared to the 10:1 ratio. According to ISO standards, the solubility of root canal sealers should not surpass 3% with our results being within these limits [26]. Nevertheless, variations in the surface-to-volume ratios of the specimens and other experimental configurations, such as the types of molds used and setting times, may contribute to the observed differences in the results compared to the other studies [39, 45, 46].

Dimensional stability of a material when placed in situ is mandatory for success of any procedure; in root canal treatment it provides integrity and hermetic seal of the root canal system, thereby minimizing the risk of microleakage and secondary infection [47]. The study results suggest that incorporating phytochemicals in 5:1 ratio may be advantageous to enhance dimensional stability since there was a significant increase in the dimensional stability of the experimental sealers as days progressed.

Satisfactory distribution of the sealer into narrow irregularities, lateral canals, and the apical foramen requires adequate flowability and an optimal film thickness. While high flow properties are desirable, they should be carefully balanced to avoid the risk of material extrusion beyond the apical foramen, which may compromise periapical healing. The American National Standards Institute (ANSI) and the American Dental Association (ADA) specifications indicate that sealers, when subjected to a flow test, should exhibit a diameter not less than 20 mm. In this study, compared to the control group, all the experimental groups showed increase in flow. AHS exhibited a higher flow of 32.47 mm and 32.42 mm compared to the other experimental sealers in both tested ratios. This could be attributed to the presence of solid paraffin hydrocarbons present in the shilajit and its likeness to be readily soluble in water but insoluble in ethanol [48, 49]. The incorporation of phytochemicals to the sealers in 5:1 ratio could be advantageous for improving the flow properties.

As per our SEM findings, all the modified sealers exhibited micro-sized particles with particle sizes ranging from 25 to 100 μm. Numerous studies support the notion that the use of smaller sealer particles can result in a thinner film thickness and enhance penetration into dentinal tubules. This could be the reason why AHS with a smaller particle size of 50 μm is found to have more flow property. The clusters observed on the surface of AHP and AHC may be attributed to the crystalline nature of the material, its immiscibility, and the increased particle size compared to the other two groups. The globular surface in AHS may result from the crystalline property of the material, given its origin from powdered rock. The smooth surface in AHA can be attributedto the smallest particle size of 25 μm and the miscibility of pachymic acid to AHR compared to other phytochemicals. Quantitative results of elements obtained through EDX microanalysis revealed the presence of sulfur and sodium in the control group, zirconium in AHP, and nickel and titanium in AHC. Furthermore, no new elements were formed in combination with the sealer and the experimental materials, confirming the integrity of AHR’s components.

FTIR is a powerful analytical technique used to characterize the chemical composition of materials. In this study, FTIR reveals strong absorption bands at approximately 749 ^cm−1^, 829 ^cm−1^, 918 ^cm−1^, 1037 ^cm−1^, 1179 ^cm−1^, 1249 ^cm−1^, 1509 ^cm−1^, and 1608 ^cm−1^that correspond to benzene derivative, presence of monosubstituted alkene, sulfoxide, and presence of an amine, strong aromatic ester, nitro compounds and the presence of unsaturated ketones. Similar findings were observed in the study by Maharti et al. [50]. Upon comparison with other samples, it is evident that the intensities of the observed peaks, such as the transmittance peak at 3459 cm-1 corresponding to -OH stretching in AHR and the peak at 1701 cm-1 related to the conjugated carbon group in AHA and AHC, are relatively low. The similarity in peaks observed in the FTIR spectroscopic data indicates that the experimental sealers maintain the integrity of AHR, with only minor modifications and trace compounds.

The XRD results of this study exhibited diffraction peaks for the various percentages of Crystalline (C) and Amorphous (A) compounds. AHR [C -11.4%, A- 88.6%], AHP [C -39.9%, A- 60.1%], AHA[C − 50.9%, A- 49.1%], AHC [C -36.5%, A- 63.5%] and AHS [C -32.6%, A- 67.4%] exhibited Calcium Tungsten Oxide (JCPDS: 41-1431). The XRD analysis also confirmed the presence of the same compound, confirming minimal or no changes in the modified sealer compared to the control. The alteration in the crystalline and amorphous composition observed in the experimental sealers in comparison to AHR could be ascribed to the miscibility of the phytochemicals. All root canal sealers showed acceptable properties based on ISO 6876 standards.

The characterization studies have affirmed that the addition of the mentioned phytochemicals to the sealer did not induce significant changes. Thus the null hypothesis was thus accepted. Therefore, the addition of 10% petasin, 0.75% pachymic acid, 0.5% Curcumin and 6% Shilajit to AH Plus sealer in the ratio 5:1 is expected to have satisfactory physicochemical properties. Despite the favorable outcomes observed in the tested materials, further investigations are necessary to gain valuable insights into the cytotoxic and anti-inflammatory behavior of this modified sealer formulation to ensure its appropriateness for clinical use.

## Conclusions

The findings of the current study indicate that the addition of 10% petasin, 0.75% pachymic acid, 0.5% Curcumin and 6% Shilajit to AH Plus sealer in the ratio of 5:1 significantly decreased the setting time and flow of AH Plus sealer, without substantially altering its dimensional stability and solubility. Therefore, further in vitro and in vivo studies on cytotoxicity and anti-inflammatory property can indicate whether these can be beneficial for application in clinical practice.

## Data Availability

The datasets used and/or analysed during the current study are available from the corresponding author.

## Abbreviations

RCS: Root canal sealer
AHR: AH Plus sealer
AHP: AH Plus sealer with petasin
AHA: AH Plus sealer with pachymic acid
AHC: AH Plus sealer with curcumin
AHS: AH Plus sealer with Shilajit
SEM: Scanning Electron Microscopy
EDS: Energy-Dispersive Spectroscopy
XRD: X-ray diffraction analysis
FTIR: Fourier-transform infrared spectroscopy
C: Crystalline
A: Amorphous

## References

[1] Hammad M, Qualtrough A, Silikas N (2009). Evaluation of root canal obturation: a three-dimensional in vitro study. J Endod.

[2] Wolf M, Küpper K, Reimann S, Bourauel C, Frentzen M (2014). 3D analyses of interface voids in root canals filled with different sealer materials in combination with warm gutta-percha technique. Clin Oral Investig.

[3] Berman LH, Hargreaves KM. Cohen’s pathways of the Pulp Expert consult - E-Book. Elsevier Health Sciences; 2015.

[4] Garrido ADB, Lia RCC, França SC, da Silva JF, Astolfi-Filho S, Sousa-Neto MD (2010). Laboratory evaluation of the physicochemical properties of a new root canal sealer based on Copaifera multijuga oil-resin. Int Endod J.

[5] Arias-Moliz MT, Ruiz-Linares M, Cassar G, Ferrer-Luque CM, Baca P, Ordinola-Zapata R (2015). The effect of benzalkonium chloride additions to AH Plus sealer. Antimicrobial, physical and chemical properties. J Dent.

[6] Ricucci D, Rôças IN, Alves FRF, Loghin S, Siqueira JF (2016). Apically extruded sealers: Fate and Influence on Treatment Outcome. J Endod.

[7] Diomede F, Caputi S, Merciaro I, Frisone S, D’Arcangelo C, Piattelli A (2014). Pro-inflammatory cytokine release and cell growth inhibition in primary human oral cells after exposure to endodontic sealer. Int Endod J.

[8] Arun S, Sampath V, Mahalaxmi S, Rajkumar K (2017). A comparative evaluation of the Effect of the addition of Pachymic Acid on the cytotoxicity of 4 different Root Canal Sealers-An in Vitro Study. J Endod.

[9] Sousa CJA, Montes CRM, Pascon EA, Loyola AM, Versiani MA (2006). Comparison of the intraosseous biocompatibility of AH Plus, EndoREZ, and Epiphany root canal sealers. J Endod.

[10] Shih Y-H, Lin D-J, Chang K-W, Hsia S-M, Ko S-Y, Lee S-Y (2014). Evaluation physical characteristics and comparison antimicrobial and anti-inflammation potentials of dental root canal sealers containing hinokitiol in vitro. PLoS ONE.

[11] Andolfatto C, Bonetti-Filho I, Carlos IZ, Guerreiro-Tanomaru JM, Kuga MC, Tormin FBC (2017). Cytocompatibility, physical properties, and antibiofilm activity of endodontic sealers with Amoxicillin. Microsc Res Tech.

[12] Ruiz-Linares M, Bailón-Sánchez ME, Baca P, Valderrama M, Ferrer-Luque CM (2013). Physical properties of AH plus with chlorhexidine and cetrimide. J Endod.

[13] Duarte MAH, Ordinola-Zapata R, Bernardes RA, Bramante CM, Bernardineli N, Garcia RB (2010). Influence of calcium hydroxide association on the physical properties of AH Plus. J Endod.

[14] Sharma A, Sanjeev K, Selvanathan VMJ, Sekar M, Harikrishnan N (2022). The evaluation of cytotoxicity and cytokine IL-6 production of root canal sealers with and without the incorporation of simvastatin: an invitro study. BMC Oral Health.

[15] Barros J, Silva MG, Rodrigues MA, Alves FRF, Lopes MA, Pina-Vaz I (2014). Antibacterial, physicochemical and mechanical properties of endodontic sealers containing quaternary ammonium polyethylenimine nanoparticles. Int Endod J.

[16] Chemical Information Review Document for Butterbur. National Toxicology Program. 2009. http://ntp.niehs.nih.gov/. Accessed 20 Jan 2024.

[17] Vinola SMJ, Karthikeyan K, Mahalaxmi S (2019). A novel petasin-modified zinc oxide eugenol sealer. J Conserv Dent.

[18] Kim T-G, Lee Y-H, Lee N-H, Bhattarai G, Lee I-K, Yun B-S (2013). The antioxidant property of pachymic acid improves bone disturbance against AH plus-induced inflammation in MC-3T3 E1 cells. J Endod.

[19] Lee Y-H, Lee N-H, Bhattarai G, Kim G-E, Lee I-K, Yun B-S (2013). Anti-inflammatory effect of pachymic acid promotes odontoblastic differentiation via HO-1 in dental pulp cells. Oral Dis.

[20] Sahebalam R, Bagheri H, Jafarzadeh H, Khodkari H, Ganjehzadeh S (2023). Tooth discoloration and solubility of Zinc Oxide Eugenol combined with different concentrations of Nano-Curcumin: an in vitro study. J Dent.

[21] Jacob A, Wu R, Zhou M, Wang P (2007). Mechanism of the anti-inflammatory effect of Curcumin: PPAR-gamma activation. PPAR Res.

[22] Aggarwal BB, Surh Y-J, Shishodia S, editors. The molecular targets and therapeutic uses of curcumin in health and disease. 2007th edition. New York, NY: Springer; 2007.

[23] Ghosal S (1990). Chemistry of shilajit, an immunomodulatory ayurvedic rasayan. Pure Appl Chem.

[24] Carrasco-Gallardo C, Guzmán L, Maccioni RB (2012). Shilajit: a natural phytocomplex with potential procognitive activity. Int J Alzheimers Dis.

[25] Sarah Victoria Ezhilarasi S, Kothandaraman R, Nesamani R, Balasubramanian S, Mahalaxmi S (2020). In vitro Assessment of cytotoxicity and anti-inflammatory properties of Shilajit Nutraceutical: a preliminary study. J Interdisciplinary Dentistry.

[26] International Organization for Standardization (2012). Dentistry - Root canal sealing materials. ISO 6876.

[27] Gatewood RS (2007). Endodontic materials. Dent Clin North Am.

[28] Johnson W, Kulild JC, Tay F, Hargreaves KM, Berman LH (2016). Obturation of the cleaned and shaped Root Canal System. Cohen’s pathways of the pulp.

[29] Langeland K (1974). Root canal sealants and pastes. Dent Clin North Am.

[30] Chauhan A, Islam AU, Prakash H, Singh S (2022). Phytochemicals targeting NF-κB signaling: potential anti-cancer interventions. J Pharm Anal.

[31] Nisar A, Jagtap S, Vyavahare S, Deshpande M, Harsulkar A, Ranjekar P (2023). Phytochemicals in the treatment of inflammation-associated diseases: the journey from preclinical trials to clinical practice. Front Pharmacol.

[32] Gonfa YH, Tessema FB, Bachheti A, Rai N, Bachheti RK (2023). Anti-inflammatory activity of phytochemicals from medicinal plants and their nanoparticles: a review. Curr Res Biotechnol.

[33] Aydın AA, Zerbes V, Parlar H, Letzel T (2013). The medical plant butterbur (Petasites): analytical and physiological (re)view. J Pharm Biomed Anal.

[34] Vinola SMJ, Karthikeyan K, Sharma A, Sudheshna S, Sekar M (2021). Anti-inflammatory efficacy of petasin-incorporated zinc oxide eugenol sealer - an in vivo zebrafish study. J Conserv Dent.

[35] Jacob A, Bernardo A, Plumitallo C, De Nuccio C, Visentin S, Minghetti L (2021). Curcumin promotes oligodendrocyte differentiation and their protection against TNF-α through the activation of the nuclear receptor PPAR-γ. Sci Rep.

[36] Ghosal S, Reddy JP, Lal VK (1976). Shilajit I: chemical constituents. J Pharm Sci.

[37] Silva EJNL, Ehrhardt IC, Sampaio GC, Cardoso ML, Oliveira Dda, Uzeda S (2021). Determining the setting of root canal sealers using an in vivo animal experimental model. Clin Oral Investig.

[38] Allan NA, Walton RC, Schaeffer MA (2001). Setting times for endodontic sealers under clinical usage and in vitro conditions. J Endod.

[39] Kamalakannan Preethi O, Sampath V, Ravikumar N, Mahalaxmi S (2020). Comparative Evaluation of Physicochemical Properties and apical Sealing ability of a Resin Sealer modified with Pachymic Acid. Eur Endod J.

[40] Koo J, Kwak SW, Kim H-C (2023). Differences in setting time of calcium silicate-based sealers under different test conditions. J Dent Sci.

[41] Baldi JV, Bernardes RA, Duarte MAH, Ordinola-Zapata R, Cavenago BC, Moraes JCS (2012). Variability of physicochemical properties of an epoxy resin sealer taken from different parts of the same tube. Int Endod J.

[42] Geurtsen W, Leyhausen G (1997). Biological aspects of root canal filling materials-histocompatibility, cytotoxicity, and mutagenicity. Clin Oral Investig.

[43] Williamson AE, Dawson DV, Drake DR, Walton RE, Rivera EM (2005). Effect of root canal filling/sealer systems on apical endotoxin penetration: a coronal leakage evaluation. J Endod.

[44] Elyassi Y, Moinzadeh AT, Kleverlaan CJ (2019). Characterization of Leachates from 6 Root Canal Sealers. J Endod.

[45] Ørstavik D, Nordahl I, Tibballs JE (2001). Dimensional change following setting of root canal sealer materials. Dent Mater.

[46] Kebudi Benezra M, Schembri Wismayer P, Camilleri J (2017). Influence of environment on testing of hydraulic sealers. Sci Rep.

[47] Yigit DH, Gencoglu N. Evaluation of resin/silicone based root canal sealers. Part i: physical properties. Digest J Nanomaterials Biostructures (DJNB). 2012;7(1).

[48] Khanna R, Witt M, Anwer K, Agarwal SP, Koch BP (2008). Spectroscopic characterization of fulvic acids extracted from the rock exudate Shilajit. Org Geochem.

[49] Kong YC, But PPH, Ng KH (1987). Chemical studies on a Nepalese panacea—shilajit (I). Int J Crude Drug Res.

[50] Maharti ID, Suprastiwi E, Agusnar H, Herdianto N, Margono A, Characterization (2023). Physical properties, and Biocompatibility of Novel Tricalcium Silicate-Chitosan Endodontic Sealer. Eur J Dent.

